# Supplementary material for: Resolving the full spectrum of human genome variation using Linked-Reads
Source: Genome Res. 2019 Apr;29(4):635–45. doi: 10.1101/gr.234443.118 (PMC6442396; doi:10.1101/gr.234443.118)
Supplement: Supplemental Material [file supp_gr.234443.118_Supplemental_longranger-2.2.zip › longranger-2.2.tar.bz2!/lr2.2-public/lib/stan/cmdstan/stan/doc/api/html/dom__err__vec_8hpp_source.html]

Stan: src/stan/math/error\_handling/dom\_err\_vec.hpp Source File


|  |  |
| --- | --- |
|  | Stan  2.5.0  probability, sampling & optimization |


- Main Page
- Related Pages
- Namespaces
- Classes
- Files

- File List
- File Members

All Classes Namespaces Files Functions Variables Typedefs Enumerations Enumerator Friends Macros Pages

- src
- stan
- math
- error\_handling

dom\_err\_vec.hpp

Go to the documentation of this file.

1 #ifndef STAN\_\_MATH\_\_ERROR\_HANDLING\_\_DOM\_ERR\_VEC\_HPP

2 #define STAN\_\_MATH\_\_ERROR\_HANDLING\_\_DOM\_ERR\_VEC\_HPP

3

4 #include <typeinfo>

5 #ifdef BOOST\_MSVC

6 # pragma warning(push) // Quiet warnings in boost/format.hpp

7 # pragma warning(disable: 4996) // \_SCL\_SECURE\_NO\_DEPRECATE

8 # pragma warning(disable: 4512) // assignment operator could not be generated.

9 // And warnings in error handling:

10 # pragma warning(disable: 4702) // unreachable code

11 // Note that this only occurs when the compiler can deduce code is unreachable,

12 // for example when policy macros are used to ignore errors rather than throw.

13 #endif

14

15 #include <sstream>

16 #include <stdexcept>

17

18 #include <boost/format.hpp>

19

20 #include <stan/math/error\_handling/dom\_err.hpp>

21 #include <stan/math/meta/value\_type.hpp>

22 #include <stan/meta/traits.hpp>

23

24 namespace stan {

25

26  namespace math {

27

28

29  // currently ignoring T\_result

30  template <typename T,

31  typename T\_result,

32  typename T\_msg>

33  inline bool dom\_err\_vec(const size\_t i,

34  const char\* function,

35  const T& y,

36  const char\* name,

37  const char\* error\_msg,

38  const T\_msg error\_msg2,

39  T\_result\* result) {

40  std::ostringstream msg\_o;

41  msg\_o << name << "[" << stan::error\_index::value + i << "] "

42  << error\_msg << error\_msg2;

43

44  std::string msg;

45  msg += (boost::format(function)

46  % typeid(typename value\_type<T>::type).name()).str();

47  msg += ": ";

48  msg += msg\_o.str();

49

50  throw std::domain\_error((boost::format(msg)

51  % stan::get(y,i)).str());

52  return false;

53  }

54

55  }

56 }

57 #endif

dom\_err.hpp

stan::math::dom\_err\_vec

bool dom\_err\_vec(const size\_t i, const char \*function, const T &y, const char \*name, const char \*error\_msg, const T\_msg error\_msg2, T\_result \*result)

**Definition:** dom\_err\_vec.hpp:33

traits.hpp

stan::error\_index::value

**Definition:** traits.hpp:20

value\_type.hpp

stan::get

T get(const T &x, size\_t n)

**Definition:** traits.hpp:143

stan::math::value\_type

Primary template class for metaprogram to compute the type of values stored in a container.

**Definition:** value\_type.hpp:21


---

[ Stan Home Page ]

*© 2011–2014,
Stan Development Team.*
